# Pregnant women’s attitudes and decision-making regarding prenatal Down syndrome screening and diagnosis: scale development and validation

**DOI:** 10.1186/s12884-020-03093-6

**Published:** 2020-07-14

**Authors:** Wei-Hsiang Huang, Shu-Fang Shih, Chen-Li Lin, Chieh-Hsing Liu

**Affiliations:** 1 Department of Health Promotion and Health Education, College of Education, National Taiwan Normal University, No. 162, Sec. 1, Heping E. Rd., Da’an Dist, Taipei City, 106 Taiwan; 2 Department of Health Management and Policy, University of Michigan, 1415 Washington Heights, Ann Arbor, MI 48109-2029 USA; 3 Department of Obstetrics and Gynecology, Taipei City Hospital, No. 12, Fuzhou St., Zhongzheng Dist, Taipei City, 100 Taiwan

**Keywords:** Down syndrome, Screening and diagnosis, Attitudes, Decision-making, Validation

## Abstract

**Background:**

Down syndrome is a common chromosomal abnormality and prenatal screening can inform parents of the risk of their baby having Down syndrome. Little research has examined how decisions regarding both Down syndrome screening as well as diagnosis are made among women who are currently pregnant and how their decisions are influenced by their social contexts, specifically family and social media, using mixed methods. The study was to test the validity and reliability of a scale that measures pregnant women’s attitudes and decision-making concerning prenatal Down syndrome screening and diagnosis in urban areas of Taiwan.

**Methods:**

We developed an item pool based on a literature review and in-depth interviews with 30 pregnant women recruited at two district hospitals in urban areas. The item pool was reviewed by a panel of experts and then administered to 300 women who had been pregnant for less than 24 weeks and had not received the Down syndrome screening tests. We used item analysis and exploratory factor analysis to validate the scale and test its reliability.

**Results:**

The initial item pool had 54 items. After the expert review, three items were deleted. After the item analysis, 16 additional items were deleted. Exploratory factor analysis of the remaining items revealed four factors labeled – “Attitudes towards Down syndrome and Screening Tests,” “Important others’ Attitudes towards Down Syndrome,” “Influence of Important Others on Decision-Making,” and “Influence of Social Media on Decision-Making” – and 16 of the remaining items had satisfactory loadings on those factors, explaining 72.0% of the total variance. The Cronbach’s α values of the dimensions ranged between 0.75 and 0.90, demonstrating satisfactory internal reliability.

**Conclusions:**

The scale has satisfactory validity and reliability, and can be used to understand pregnant women’s attitudes and decision-making regarding Down syndrome screening and diagnosis, and to help design tailored consultations for pregnant women in clinical settings.

## Background

Down syndrome is a common chromosomal abnormality and is associated with various comorbidities, such as congenital heart disease, gastrointestinal disorders, immunodeficiency, visual and hearing impairment, skeletal dysplasia, physical growth delay, and intellectual disability [1]. Prenatal screening can inform parents of the risk of their baby having Down syndrome. Invasive diagnostic tests such as Amniocentesis or Chorionic Callus sampling involve chromosomal testing can be performed to confirm Down syndrome in fetuses [2].

Down syndrome screening is noninvasive and the test results reveal the risk of fetuses having Down syndrome. When the screening test result is positive, a pregnant woman needs to decide whether to receive further diagnostic tests and possibly terminate her pregnancy if the diagnostic test result is positive [3, 4].A false positive may result in invasive diagnostics or unnecessary anxiety in pregnant women, both of which increase the risk of miscarriage [3, 5, 6]. Studies have shown that women are unable to distinguish between various Down syndrome screening tests and they are not fully informed of the benefits and risks associated with those tests [3, 7]. As a result, pregnant women often have difficulty deciding whether to receive screening and diagnostic tests [8–10], particularly when the decision must be made within a limited time period [6]. Most pregnant women therefore rely on healthcare professionals to provide unbiased and accurate information to help them make decisions [11]. Yet, the technical nature of different Down syndrome screening tests are difficult to explain and may cause confusion and anxiety in pregnant women [12]. A meta-synthesis suggested a need for research that focused on examining pregnant women’s experiences and decision-making about screening for Down syndrome [13].

Two Down syndrome screening tests are commonly recommended by OB/GYN physicians for the routine prenatal care in Taiwan [14]: one in the first trimester (weeks 11–14) and the other the second trimester (weeks 15–20). The first trimester combined test involves using ultrasound to examine a fetus’ nuchal translucency (NT), along with the test of maternal serum markers (free β-hCG and PAPP-A). It is able to detect 82–87% of fetuses with Down syndrome [15], The second trimester quadruple screening measures maternal serum markers (free β-hCG, AFP, uE3, and inhibin A) and detects 60–83% of fetuses with Down syndrome. According to the most recent study, the first-trimester screening had higher performance with a lower false positive rate than the second-trimester screening [16]. If the maternal serum marker test indicates a higher than 1-in-270 risk of Down syndrome, the pregnant woman is advised to receive an invasive procedure, such as Amniocentesis or Chorionic Villus Sampling, as a diagnosis test.

Because of the prevalence of Down syndrome, a multitude of studies examined mothers’ knowledge, beliefs, and attitude about the abnormality. In particular, many studies focused on decision making following a prenatal diagnosis of Down syndrome using survey [17, 18]or used qualitative interview to explore the decision-making process termination of pregnancy following a prenatal diagnosis [19] or focused only on those who had higher risk of fetal Down syndrome [20]. One qualitative research focused on describing the circumstances in which maternal serum screening for Down syndrome was offered to pregnant women and their reasons for undertaking it in Taiwan [21] and one study used meta-synthesis approach to report qualitative research studies related to pregnant women’s decision-making process only with regards to antenatal screening for Down syndrome [13]. To the best of our knowledge, little research has examined how decisions regarding Down syndrome screening as well as diagnosis are made among women who are currently pregnant but have not yet receiving any Down syndrome screening or diagnostic tests in Taiwan, and how their decisions are influenced by their social contexts, specifically family and social media in other countries. This paucity of research is likely due to the fact that no valid scale is available to assess pregnant women’s attitude and decision-making regarding Down syndrome screening and diagnosis. Moreover, a valid scale may help health providers to assess, in a patient and family-centered way, how pregnant women approach the choice of Down syndrome screening and diagnosis and to assist pregnant women in making decisions that are uncertain and may have significant consequences to their families. The purpose of this study, therefore, was to develop and validate a scale that measured attitude and decision-making regarding prenatal Down syndrome screening and diagnosis among pregnant women who had not received screening in urban areas of Taiwan. In developing and validating the scale, we drew on a hospital-based sample of pregnant women in Taipei City, where the cost of Down syndrome screening and diagnosis is low because of government subsidies. This allowed us to exclude cost as a consideration and focus pregnant women’s attitudes and their social contexts in decision-making.

## Methods

This study employed a cross-sectional design and was conducted between March 2016 and December 2017 in two district public hospitals in Taipei, Taiwan. We used a mixed-methods approach – specifically, an exploratory sequential design [22] – to develop and validate a scale to assess pregnant women’s attitudes and decision-making regarding Down syndrome screening and diagnosis tests. Our design involved the following steps. First, we conducted a broad review of literature to search for existing scales or survey items in relation to attitudes and decision-making of Down syndrome screening and diagnosis tests. Second, we performed in-depth interviews with 30 women who had their prenatal care visits at these two district public hospitals, were in first or second trimester of pregnancy, and who had not received Down syndrome screening. All the interviews were audio-recorded and transcribed. We analyzed the transcriptions to identify themes and issues of importance to pregnant women. Third, we developed an item pool based on the results of government guidelines, literature review and in-depth interviews [23, 24]. The initial pool consisted of 54 items, related to pregnant women’s attitudes towards Down syndrome and Down syndrome screening, factors affecting pregnant women’s Down syndrome screening and diagnosis decisions, and perceptions of family members’ and friends’ views of Down syndrome. Fourth, we conducted a Delphi process with four panelists with expertise in Obstetrics and Gynecology, Nursing, and Behavioral Sciences to review the item pool. To establish expert’s validity of the instrument, the experts were asked to rate the relevance (whether the item is important to be included), appropriateness (whether the item is appropriately worded), and clarity (whether the item is clear and easily understandable) of each item on a 4-point Likert scale. A higher rating meant higher relevance, appropriateness, or clarity. Panelists were expected to suggest wording changes for items that received low ratings. We calculated the Content Validity Index (CVI), a widely used index for measuring the degree to which an item is appropriate for the construct being measured, for each item by averaging the scores by the four panelists and deleted 3 items that had a CVI less than 2. The remaining 51 items were revised, if needed, and sent back to the panelists for another review before they were finalized for validation.

Finally, we conducted a survey to validate the 51 items using a convenient sample of pregnant women who attended the prenatal care visits at the two district public hospitals in Taipei City. Survey participants were women who had been pregnant for less than 24 weeks and had not received Down syndrome screening by the time of the survey. We excluded those who were medically eligible to receive a diagnosis test or who wished to receive a diagnosis test without a screening test first. In accordance to Wu & Tu (2012), the sample size for validation should be at least five times the number of items to be validated [25]. With a pool of 51 items, the minimal sample size should be 255. To reach this target number and assuming a 80% response rate, we recruited 320 pregnant women to participate in the survey; 300 agreed and provided valid responses, achieving a valid response rate of 93.8%. The average age of participants was 32.5, with a range between 21 and 44. The very majority (83.0%) of participants had a college level education or above and 17.0% had a high school diploma. Close to three-fourths (74.8%) worked full-time and the remainder worked part-time or were unemployed. About 11% had a monthly household income of less than NT$30,000, 15.5% between NT$30,000 and NT$50,000, 15.9% between NT$50,000 and NT$70,000, 29.8% between NT$70,000 and NT$100,000, and 25.3% over NT$100,000. About 66% have a religion. Approximately 8% reported that they were a smoker before pregnancy, and 2.3% reported that they were still smoking during pregnancy. Very few (3.0%) reported that they had poor physical health, 39.6% fair, and 57.8% good or excellent physical health. Similarly, very few (4.3%) reported having poor mental health, 44.0% fair, and 51.6% good or excellent mental health. About 25% had chronic conditions. About half of the participants (51%) were first-time mothers, 40.0% were having their second baby when interviewed; about 9.3% were having their third or more babies when interviewed. Close to 70% reported that the pregnancy were unintended. A few (10.0%) women reported that they knew children with Down syndrome; only 4.0% had a genetic consultation; 1.3% reported that they had family members with Down syndrome, and 5.3% were not sure. Only about 35% of women had ever received Down syndrome screening information.

To validate the 51 items, we performed a series of analyses, including item analysis, exploratory factor analysis (EFA), and internal consistency analysis (the Cronbach’s α). The purpose of item analysis was to examine the quality of each item and to retain items that showed a strong correlation with other items and that were able to differentiate the levels (i.e., high versus low) of attitudes and decision-making among pregnant women. EFA helped to uncover the underlying, latent structure of retained items. We used Principal Component analysis to extract factors with an eigenvalue higher than 1 and performed Varimax Orthogonal Rotations in the analysis. Finally, we calculated the Cronbach’s α value of retained items to examine their reliability to assure the items have good internal consistency. All statistical analyses were performed using IBM SPSS for Windows 22.0.

## Results

### Item analysis

Table 1 displays results of the item analysis. The second column shows the correlations between individual items and the total score of 51 items retained after the Delphi process. It was expected that if a participant responded positively to an item, she should, in general, have a higher overall score than participants who responded not as positively to an item. Sixteen of the items had a correlation coefficient of less than 0.30, which based on Wu and Tu (2012) may be too low and therefore may be candidates for deletion [25]. The third column shows the Cronbach’s α value that would result should an item was deleted. In comparison to the Cronbach’s α for all 51 item (0.724), removing three items would have produced a higher Cronbach’s α value (i.e., 0.726, 0.726, and 0.725, respectively) and improved the item pool’s internal consistency [25]. Finally, the last column displays each item’s critical ratio (CR), which indicated the degree to which the item was able to differentiate between participants with a high level of response to the item pool vis-à-vis participants with a low level of response. A statistically significant CR value means the item has a satisfactory differentiation power. The CR values of 6 items did not reach a significant level.

**Table 1:** Item Analysis Results

No.	Item	Correlation between items and the total score	Cronbach’s α after deletion (Original α = 0.724)	CR
1	A1	0.291	0.721	3.452*
2	A2	0.120	0.723	1.527
3	A3	0.415	0.720	5.872*
4	A4	0.396	0.720	5.544*
5	A5	0.346	0.720	4.655*
6	A6	0.349	0.719	6.148*
7	A7	0.388	0.720	6.093*
8	A8	0.421	0.720	6.119*
9	A10	0.373	0.720	5.651*
10	A11	0.296	0.720	4.847*
11	A12	0.295	0.720	3.884*
12	A13	0.308	0.720	4.882*
13	A14	0.468	0.717	7.709*
14	A15	0.112	0.724	1.892
15	A16	0.430	0.719	7.086*
16	A17	0.221	0.722	2.712
17	A18	0.452	0.717	7.823*
18	A19	0.270	0.721	3.101*
19	A20	0.342	0.721	3.849*
20	A21	0.467	0.717	7.240*
21	A22	0.458	0.719	6.813*
22	A23	0.532	0.716	9.598*
23	A24	0.520	0.716	8.421*
24	A25	0.499	0.716	7.487*
25	A26	0.533	0.716	8.186*
26	A27	0.556	0.716	8.411*
27	A28	0.270	0.703	7.739*
28	A29	0.345	0.719	4.796*
29	A30	−0.033	0.726	0.348
30	A31	0.343	0.720	4.808*
31	A32	0.243	0.721	4.312*
32	A33	0.236	0.721	3.585*
33	A34	0.370	0.718	5.581*
34	A37	0.388	0.718	4.297*
35	A38	0.265	0.721	4.280*
36	A39	0.338	0.720	5.713*
37	A40	0.498	0.717	7.568*
38	A41	0.488	0.718	6.710*
39	A42	0.542	0.716	10.205*
40	A43	0.491	0.716	8.611*
41	A44	0.533	0.716	9.075*
42	A45	0.452	0.717	6.406*
43	A46	0.444	0.718	6.059*
44	A47	0.281	0.719	5.561*
45	A48	0.248	0.721	4.385*
46	A49	0.310	0.720	5.286*
47	A50	0.119	0.726	0.586
48	A51	0.142	0.725	0.951
49	A52	0.385	0.718	6.415*
50	A53	0.502	0.716	8.285*
51	A54	0.477	0.716	7.899*

Based on results of the item analysis, we decided to remove 16 items (highlighted in gray in Table 1) and retain 35 items in the EFA.

### Exploratory factor analysis (EFA)

We performed the Kaiser–Meyer–Olkin (KMO) test to verify if our data were suitable for factor analysis. KMO returns value ranging between 0 and 1; the higher the value, the more suitable the data are for factor analysis. Specifically, a value between 0.8 and 1 indicates adequate suitability and factory analysis is meritorious [26]. The KMO value in our study sample was 0.829. In addition, we performed the Bartlett’s test of sphericity, which aims to test the correlations among items. The analysis showed that the approximate Chi-Squared value was 5658.80 with *p* < 0.001, indicating our data were suitable for factor analysis.

We performed five rounds of EFA and used the following 4 criteria for item deletion: (1) items of factors with less than 3 items [27]; (2) items with a factor loading less than 0.5 [28]; (3) items loaded on more than one factor [29]; and (4) items loaded on unintended factors [30].

The first round of EFA identified 9 factors that had an eigenvalue higher than 1 and that together explained 67.74% of the total variance; those items were deleted in accordance with the first criterion for deletion. Six additional items were also deleted because they loaded on more than one factor. In total, 10 items were deleted during in the first round of EFA.

The second round of EFA found 7 factors that had an eigenvalue higher than 1. Together, they explained 69.63% of the total variance. One factor was loaded by 2 items; those items were deleted. Two items loaded on more than one factor and were deleted. This round of EFA, thus, resulted in the deletion of 4 items.

Results of the third round of EFA showed that five factors had an eigenvalue higher than 1 and explained 67.32% of the total variance. One factor was loaded with fewer than three items and those items were deleted. One item loaded onto more than one factor and it was deleted. In total, three items were deleted.

We performed the fourth round of EFA, which identified 4 factors with an eigenvalue higher than 1, explaining 66.68% of the total variance. All of the factors were loaded with at least three items; the factor loadings of the item were all greater than 0.5 and no item was loaded on more than one factor. However, two items were loaded on unintended factors; therefore, we deleted these two items and performed the EFA again. We finally identified 4 factors with an eigenvalue higher than 1, explaining 71.97% of the total variance. The factor loadings of the item were all greater than 0.5; no item was loaded on more than one factor; and all items were loaded on intended factors. Although one factors have only two items, researchers reached the consensus on keeping this factor because of its importance. The KMO value was 0.82, indicating satisfactory suitability for a factor analysis (KMO falls between 0.8 and 0.9 means satisfactory) [26]. The approximate Chi-Squared value in the Bartlett’s test of sphericity was 2816.20 (*p* < 0.001).

After conducting EFA, our study identified four factors and we labeled them: “Attitude towards Down Syndrome and Screening tests” (six items), "Attitudes of Important Others^1^ toward Down Syndrome" (two items), “Influence of Important Others on Decision-Making” (five items), and “Influence of Social Media on Decision-Making” (three items). The scale called Down syndrome Attitudes and Decision-Making towards Screening and Diagnosis (DS-ADMSD), had a total of 16 items (detailed in the Additional file 1). Table 2 summarizes the results of the EFA.

**Table 2:** Results of the EFA

Question No. /original item	Attitudes towards Down Syndrome & Screening tests	Important others’ Attitudes towards Down Syndrome	Influence of Important others on Decision Making	Influence of Social Media on Decision Making	Communality
1/A4	0.881	0.099	−0.011	0.004	0.786
2/A3	0.876	0.078	0.009	0.005	0.773
3/A5	0.866	−0.002	0.005	0.035	0.751
4/A8	0.856	0.005	0.004	0.027	0.734
5/A7	0.829	0.026	0.015	−0.020	0.688
6/A6	0.648	0.049	0.004	0.144	0.443
7/A53	0.098	0.916	0.048	0.073	0.857
8/A54	0.067	0.894	0.166	0.086	0.838
9/A24	0.010	0.031	0.914	0.195	0.875
10/A25	−0.025	0.014	0.904	0.185	0.852
11/A23	−0.011	0.063	0.769	0.330	0.705
12/A21	0.030	0.140	0.769	0.003	0.611
13/A44	0.003	0.062	0.699	0.373	0.631
14/A45	0.002	0.049	0.190	0.837	0.739
15/A46	0.026	0.026	0.223	0.746	0.608
16/A26	0.150	0.114	0.286	0.712	0.625
Eigenvalue	4.701	1.233	1.044	1.537	
Items maintained	6	2	5	3	
Variance explained	26.071	22.010	13.254	10.638	
Cumulative variance explained	26.071	48.081	61.335	71.973	
KMO		0.822			
Bartlett’s test of sphericity (χ^2^)		χ^2^ = 2816.198, *p* < .001			

All dimensions were rated on a 5-point Likert scale (Strongly disagree, Disagree, Neither Agree or Disagree, Agree, and Strongly agree). Other information for each dimension is detailed as follows:

1. Attitudes towards Down Syndrome and Screening Tests.
  A total of six items in this dimension. The higher the total score is, the more positive a pregnant women’s attitude toward Down syndrome screening.
2. Attitudes of Important Others towards Down syndrome.
  This dimension comprised two items. The higher the total score is, the more negative attitudes of a pregnant woman’s family toward having a Down syndrome baby.
3. Influence of Important Others on Decision-Making.
  This dimension comprised five items. The higher the total score is, the stronger the influence of important others on the choice of a pregnant woman regarding screening tests and diagnosis.
4. Influence of Social Media on Decision-Making.

This dimension comprised three items. The higher the total score is, the stronger the influence of social media (such as online information or other Internet users) on a pregnant woman’s decision-making regarding means of diagnosis.

### Reliability test

This study conducted a reliability test on the scale, which revealed that the four factors have good internal consistency (Cronbach’s α). According to the test results in Table 3, the Cronbach’s α for four factors were 0.898, 0.897, 0.747, and 0.818, respectively. The overall internal consistency (Cronbach’s α) was 0.832.

**Table 3:** Reliability Test of the Scale

Factors	Cronbach’s α coefficient
Attitudes Toward Down syndrome and Screening Tests	0.898
Important other’s Attitudes towards Down syndrome	0.818
Influence of Important others on Decision-Making	0.897
Influence of Social Media on Decision-Making	0.747
Overall scale	0.832

## Discussion

Over the past decades, many scales have been developed to measure patients’ attitudes toward health screening. However, little research has been done in relation to pregnant women’s attitudes and decision making specifically toward Down syndrome both screening and diagnosis in Taiwan and other countries. This study was the first in Taiwan to develop and validate a scale concerning pregnant women’s attitudes and decision-making regarding Down syndrome screening and diagnosis. We adopted a mixed-methods design, which included qualitative interviews to better understand the pregnant women’s perceptions about Down syndrome screening and diagnosis and developed an initial item pool, a review of the item pool by experts to establish the face validity, a survey of pregnant women to collect quantitative data for the item validation, and a psychometric analysis to validate and select items to be included in the final scale. The results showed the final scale, which included 16 items, exhibited satisfactory validity and reliability.

Studies have developed related scales and verified their reliability and validity [21, 23, 24]. These scales focused on informed choice, rather than the attitude and decision-making of pregnant women, concerning Down syndrome screening tests. Furthermore, those previous studies pertained primarily to Down syndrome screening or diagnosis. In contrast, we considered Down syndrome screening and diagnosis as interconnected issues on a continuum of decision-making faced by pregnant women and their families and incorporated both elements in the scale development.

Our study showed that pregnant women’s attitudes and decision-making towards Down syndrome screening and diagnosis were multi-dimensional; the items on the final scale could be categorized into four related yet separate dimensions, namely “Attitudes towards Down Syndrome and Screening Tests,” “Attitudes of Important Others towards Down Syndrome,” “Influence of Important Others on Decision-Making,” and “Influence of Social Media on Decision-Making.” Consistent with the findings from previous qualitative studies on understanding decision-making processes with regard to prenatal screening for Down syndrome [13], the dimensions of our scale – specifically those in relation to women’s attitudes and influence of their important others – reflect personal values and emotional support, which were found to be significant factors for women’s choices of prenatal screening tests. The dimensions also reflect the culture and tradition in Chinese society where childbearing is not only a vital life event for a couple, but also an important social milestone for their families and close social circle.

Studies have shown that the Internet and social media have become the most popular sources of health information for pregnant women [31]. Similarly, pregnant women in our sample also relied on the online and social media for information and advice regarding prenatal screening and diagnostic tests. Therefore, most pregnant women may learn the knowledge about Down syndrome screening and diagnostic tests, experience shared by the online communities from the Internet. In addition, pregnant women in Taiwan usually perceived prenatal screening as routine care and most of them prefer not to have children with Down syndrome if the likelihood of having a Down syndrome children is high, women might just chose to receive diagnostic test based on the screening test results without consulting their healthcare providers. Nevertheless, regardless of how convenient the Internet information is, it is not all accurate, and pregnant women may not have the ability to judge the quality of the online information. In Taiwan, the government has provided brochures and websites to educate pregnant women on Down syndrome and its screening and diagnosis. It is unclear if, and to what extent, such official education materials constitute the main source of information for women. It would be important to understand the usual channels through which pregnant women access the information, their ability to determine the accuracy of the information, and how the different sources of information affect their choices with regard to Down syndrome screening and diagnosis.

Our final scale did not include items related to cost, convenience, age, and family history. As we have explained before, the Taipei municipal government has subsidized Down syndrome screening. Moreover, due to the abundance of health care resources in the city, cost and convenience may not be a concern to the pregnant women who participated in the study. The subsidy and wide availability of health care resources – thus the convenience of tests – may have made Down syndrome screening and diagnosis commonplace among pregnant women in Taipei, which explains the absence of items related to age and family history on our scale. It is possible that these factors – cost, convenience, age, and family history – are important considerations for pregnant women in areas, countries, and regions where financial and geographic access to Down syndrome screening and diagnosis is limited, and where the decision for receiving Down syndrome screening and diagnosis tests are deemed unusual. If so, future research is needed to extend our work and adapt the scale to different socioeconomic and cultural contexts.

Previous research on the screening and diagnosis of Down syndrome has largely overlooked the influence of important others, particularly that of a spouse, significant other, family members, and friends. The dimensions on our scale suggest that a pregnant woman’s decisions in relation to Down syndrome screening and diagnosis may be strongly influenced by her important others. Our scale, therefore, may uncover different aspects of a pregnant women’s decision-making and thereby help clinicians to provide tailored and patient and family-centered consultations to pregnant women who have to weigh difficult decisions about Down syndrome screening and diagnosis. Considering the importance of social influence and support, it may be useful to focus health education and counseling efforts on important others – e.g., spouse, significant other, family members – in addition to the pregnant women. Broad engagement of important others may help to ensure strong support for pregnant women during a time of potentially difficult decision-making. Whether to engage important others in the health education and counseling processes should be the pregnant women’s decision, however, and research is needed to understand if, and under what conditions, the involvement of important others may produce undue pressure on the pregnant women.

The scale we have developed contains only 16 items, and it is easy to use and has good validity and reliability. Clinicians and researchers can use it to assess pregnant women’s attitudes and decision-making regarding Down syndrome screening and diagnosis. Applied in a clinical setting, the scale will enable clinicians to provide pregnant women with tailored information and help them to make informed decisions based on the results of this scale. Due to the limited amount of time clinicians usually spend with pregnant women in Taiwan, how to assess their attitudes in an efficient manner and provide just-in-time education or intervention has become critically important. We anticipate that the availability of the scale may also increase research efforts to better understand the conditions that facilitate or inhabit pregnant women and their family to make informed choices regarding Down syndrome detection.

Many decision aids have been applied in the clinical field to help people make informed decisions. They include informing people about the decisions they have to make, providing related benefits and risks, and helping them to clarify their values [32, 33]. Studies have found that that decision aids in prenatal tests increased pregnant women’s knowledge, reduced conflicts in decision-making, and alleviated anxiety. According to a recent study analyzing factors influencing pregnant women’s use of a patient decision aid for deciding about prenatal screening for Down syndrome, one of the most frequent-mentioned domains is that opinion of the pregnant women’s partner in addition to patient decision aids [34], therefore, the scale we developed could provide a tool to understand pregnant women’s attitudes and decision-making process before using the decision aids.

A major limitation is the study sample, which consisted of pregnant women recruited from two public district hospitals in Taipei City. The study sample may over-represent women with middle and higher socioeconomic status. The scale would therefore need further validation from samples with a broader socioeconomic and geographic representation.

## Conclusions

Our study suggests that the scale has satisfactory validity and reliability, and can be used to understand pregnant women’s attitudes and decision-making regarding Down syndrome screening and diagnosis, and to help design tailored consultations for pregnant women in clinical settings. In addition, researchers can use this scale to help design tailored messages in decision aids. Using the scale in clinical setting can provide healthcare providers to understand pregnant women’s attitudes and decision-making process before use decision aid tools, which can assist pregnant women in making decisions related to screening tests. In addition, healthcare providers could also provide tailored education and consultation to pregnant women. Tailored education and information may empower women and assist them in making informed, sound decisions that would benefit their health as well as the health of their children.

## Supplementary information

**Additional file 1.** Survey instrument (Down syndrome Attitudes and Decision-Making towards Screening and Diagnosis)

## Data Availability

The datasets generated and/or analyzed during the current study are not publicly available due to the regulations of Institutional Review Board of Taipei City Hospital but are available from the corresponding author on reasonable request.

## Abbreviations

OB/GYN: Obstetrics and Gynecology
NT: Nuchal Translucency
free β-hCG: Free β-Human chorionic gonadotropin
PAPP-A: Pregnancy-associated plasma protein-A
AFP: Alpha-fetoprotein
uE3: Unconjugated estriol
CVI: Content Validity Index
NT$: New Taiwan dollar
US$: United States Dollar
EFA: Exploratory Factor Analysis
PCA: Principal Component Analysis
IBM SPSS: International Business Machines Corporation Statistical Product and Service Solutions
CR: Critical ratio
KMO: Kaiser–Meyer–Olkin

## Footnotes

1 Important others refer to spouse, significant other, family members, relatives, and friends.
