# Supplementary material for: Pregnant women’s attitudes and decision-making regarding prenatal Down syndrome screening and diagnosis: scale development and validation
Source: BMC Pregnancy Childbirth. 2020 Jul 14;20:407. doi: 10.1186/s12884-020-03093-6 (PMC7362405; doi:10.1186/s12884-020-03093-6)
Supplement: Supplementary file 1 — Additional file 1. Survey instrument (Down syndrome Attitudes and Decision-Making towards Screening and Diagnosis) [file 12884_2020_3093_MOESM1_ESM.docx]

**Down syndrome Attitudes and Decision-Making towards Screening and Diagnoses (DS-ADMSD)**

Please rate your level of agreement with the following statements:

| **Questions** | **Strong**  **Agree** | **Agree** | **Neither**  **Agree**  **Or**  **Disagree** | **Disagree** | **Strongly**  **Disagree** |
| --- | --- | --- | --- | --- | --- |
| **Attitudes towards Down Syndrome** | | | | | |
| 1. Receiving Down syndrome screening is beneficial to me. |  |  |  |  |  |
| 2. Down syndrome screening is important. |  |  |  |  |  |
| 3. Down syndrome screening is a test that I would like to take. |  |  |  |  |  |
| 4. Down syndrome screening assures me by helping me understand my baby’s health conditions. |  |  |  |  |  |
| 5. Down syndrome screening helps me learn about my baby’s conditions. |  |  |  |  |  |
| 6. Knowing the risk level from Down syndrome screening helps me decide whether to receive a diagnosis test. |  |  |  |  |  |
| **Important others’ Attitudes towards Down Syndrome** | | | | | |
| 7. My family cannot accept a child with Down syndrome. |  |  |  |  |  |
| 8. My family members, relatives, and friends would never accept me having a child with Down syndrome. |  |  |  |  |  |
| **Influence of Important others on Decision Making** | | | | | |
| 9. I follow my spouse’s advice when choosing a Down syndrome screening test. |  |  |  |  |  |
| 10. I follow my parents’ (parents-in-law included) advice when choosing a Down syndrome screening test. |  |  |  |  |  |
| 11. In general, I follow my family members’ advice when choosing a Down syndrome screening test |  |  |  |  |  |
| 12. I follow my friends’ advice when choosing a Down syndrome screening test. |  |  |  |  |  |
| 13. I follow my parents’ (parents-in-law included) advice in deciding on an invasive test to diagnose Down syndrome. |  |  |  |  |  |
| **Influence of Social Media on Decision Making** | | | | | |
| 14. I rely on online health education information when choosing a Down syndrome screening test. |  |  |  |  |  |
| 15. I rely on online health education information in deciding on an invasive test to diagnose Down syndrome. |  |  |  |  |  |
| 16. I follow online peers’ advice in deciding on an invasive test to diagnose Down syndrome. |  |  |  |  |  |
